# Association between Functional Connectivity of Entorhinal Cortex and Olfactory Performance in Parkinson’s Disease

**DOI:** 10.3390/brainsci12080963

**Published:** 2022-07-22

**Authors:** Wentao Fan, Hui Li, Haoyuan Li, Ying Li, Jing Wang, Xiuqin Jia, Qi Yang

**Affiliations:** 1 Department of Radiology, Beijing Geriatric Hospital, Beijing 100095, China; wtfan2019@163.com; 2 Department of Radiology, Beijing Chaoyang Hospital, Capital Medical University, Beijing 100020, China; lihui_9025@163.com (H.L.); shucklelhy@126.com (H.L.); xqjia2014@163.com (X.J.); 3 Department of Radiology, Beijing Anzhen Hospital, Capital Medical University, Beijing 100029, China; liying_04@126.com; 4 Department of Clinical Lab, Beijing Chaoyang Hospital, Capital Medical University, Beijing 100020, China; kyc6636@126.com; 5 Key Lab of Medical Engineering for Cardiovascular Disease, Ministry of Education, Beijing 100020, China

**Keywords:** Parkinson’s disease, olfactory performance, entorhinal cortex, resting-state functional MRI, functional connectivity

## Abstract

The present study aimed to investigate the association between the functional connectivity (FC) of the olfactory cortex and olfactory performance in Parkinson’s disease (PD). Eighty-two early PD patients and twenty-one healthy controls underwent structural and resting-state functional MRI scans, as well as neuropsychological assessments from the Parkinson’s Progression Markers Initiative database. A whole brain voxel-wise regression analysis was conducted to evaluate the relationship between the FC of the entorhinal cortex (EC-FC) and olfactory performance. Then, a one-way ANCOVA, based on the regions of interest, was performed with SPSS to investigate the group differences and correlation analysis that were used to analyze the relationships between the FC and neuropsychological assessments. In addition, regression models were used to evaluate the risk factors for the decreased olfactory function. A significantly negative correlation was observed between the olfactory performance and the left EC-FC in the right dorsal cingulate gyrus (dCC) in patients. The PD patients with anosmia exhibited significantly higher FC values than the PD patients with normal olfaction or the PD patients with mild to moderate microsomia. Except for the olfactory performance, no significant correlation was detected between the neuropsychological assessments and the FC values. A linear regression analysis revealed that the increased FC and Geriatric Depression Scale are independently associated with lower the University of Pennsylvania Smell Identification Test scores. The current findings enhanced the understanding of olfactory dysfunction-related pathophysiological mechanisms in early PD and suggested that the left EC-FC in the right dCC may be a potential neuroimaging biomarker for olfactory performance.

## 1. Introduction

Olfactory dysfunction (OD) is frequently implicated at the prodromal stage of Parkinson’s Disease (PD) [1,2]. Still, its pathophysiological mechanism is unclear. Olfactory testing has been used to identify the early-stage of PD [3]. However, high prevalence of OD in normal aging and the unclear interaction between olfactory loss and developed PD lead to the unsatisfactory results of olfactory tests in detecting PD [4,5,6]. Thus, it is of great significance to find a neuroimaging biomarker specific to early PD, which may provide diagnostic and therapeutic values before the onset of motor symptoms.

Parkinson’s disease with olfactory dysfunction (PD-OD) has been studied using task-functional magnetic resonance imaging (fMRI). However, these findings related to the olfactory cortex are inconsistent. For example, compared with healthy controls (HCs), some studies showed decreased activity in the cerebral olfactory system in PD [7,8,9], and one study found reduced activation of the olfactory cortex at PD’s early stage [10]. In contrast, another study reported no significant differences in the olfactory cortex in PD [11]. These inconsistencies might be attributed to different task designs to some extent. For example, Hummel et al. used either unpleasant or pleasant stimuli, while Georgiopoulos et al. utilized coffee oil and vanillin [8,11]. By comparison, resting-state functional MRI (rs-fMRI) has advantages over task-fMRI without deliberate stimulation or intentional movement involved [12,13], which could avoid the confounding effect of different tasks.

So far, there are few studies using rs-fMRI that examined spontaneous activity in PD patients’ olfactory cortex. EC is one of the important primary olfactory areas which are receiving direct olfactory input from the olfactory bulb [14]. Meanwhile, EC is the “gate” for sensory information from many cortices to enter the hippocampus. A study reported the direct lateral EC→dCA1 (dorsal Cornu Ammonis) circuit is the key center for olfactory associative learning [15]. According to previous research, the EC is a critical region for olfactory function. In addition, the association between the EC and olfactory performance in PD patients is still poorly understood and not fully investigated. Taken together, the neural activity of EC in PD patients was specifically of interest in the current study. We aimed to investigate the association between EC-FC and olfactory performance using whole brain voxel-wise regression analysis. It was hypothesized that the EC-FC would be disrupted and associated with the severity of hyposmia in PD.

## 2. Materials and Methods

### 2.1. Participants

Data used in our study came from the Parkinson’s Progression Markers Initiative (PPMI) (http://www.ppmi-info.org/data, accessed on 1 December 2020) [16]. All PD patients met the following criteria (1) have more than one symptom such as rigidity, bradykinesia, and resting (or have asymmetric bradykinesia or asymmetric resting tremor); (2) Hoehn and Yahr (H&Y stage) I–II at baseline; (3) SPECT images show dopamine transporter dysfunction. The following types of PD patients were further excluded from the current study: (1) any history of nasal and psychiatric or neurological disease; (2) PD patients accompanied other PD-associated comorbid conditions that significantly affect olfactory function. The HCs were included by the following criteria: (1) no first-degree member with idiopathic PD; (2) no current or active clinically diagnosis of neurologic diseases; (3) Montreal Cognitive Assessment (MoCA) score of more than 26. The ethical standards committee approved the study at each PPMI site. Written informed consent was obtained from all patients. A total of 82 PD patients (63 men, 61.5 ± 10.4 years of age) and 21 HCs (16 men, 61.2 ± 10.3 years of age) were recruited. They underwent both structural T1 MRI and rs-fMRI scanning at the same time.

### 2.2. The Clinical and Neuropsychological Rating Scale

All PD patients underwent Hoehn and Yahr staging (H&Y stage), Unified Parkinson’s Disease Rating Scale (UPDRS), Modified Schwab and England Activities of Daily Living (ADL), and Scales for Outcomes in Parkinson’s disease-Autonomic (SCOPA-AUT).

To measure the neuropsychological state, all participants were administered the MoCA for global cognition function, the Semantic Fluency total score (SF) and the Letter-Number Sequencing (LNS) for verbal fluency, the Hopkins Verbal Learning Test-Revised (HVLT) for recall and recognition, the Symbol Digit Modalities score (SDMT) for attention, and the Benton Judgment of Line Orientation Score (JLO) for visuospatial ability. Depression was assessed by the Geriatric Depression Scale (GDS) and the State-Trait Anxiety Index (STAI).

For all participants, the olfactory performance was assessed using the University of Pennsylvania Smell Identification Test (UPSIT). It’s a ‘scratch-and-sniff’ odor identification test consisting of the full 40 items [17]. Based on normative UPSIT scores, which were normalized for sex and age [18], we divided PD patients into four groups: the UPSIT scores of above 33 were classified as PD-normal olfaction, and scores <19 were considered to reflect anosmia. PD patients with mild to moderate or severe macrosomia with the UPSIT scores between 25 and 33 and 19–25, respectively [19]. All clinical characteristics and neuropsychological assessments of participants were presented in Table 1.

### 2.3. MRI Data Acquisition

MRI data were acquired using 3.0 Tesla Siemens Scanner (Trio system). Functional MRI data were acquired with the parameters of TR/TE 2400 ms /25 ms, 40 slices with 3.3 mm thickness, FOV 240 × 240 mm^2^, 68 × 66 matrix dimension, and 80° flip angle. High-resolution structural images were obtained with these parameters of TR/TE 2300 ms/2.89 ms, 176 sagittal slices with 1-mm thickness, 256 × 240 matrix size, and 9° flip angle. FMRI data were obtained by using an EPI sequence that lasted 7 min (210 volumes).

### 2.4. Rs-fMRI Preprocessing and Functional Connectivity Analysis

Rs-fMRI data preprocessing was performed using SPM12 (http://www.fil.ion.ucl.ac.uk/spm, accessed on 1 December 2020), and seed-to-voxel correlation analysis was performed by the FC (CONN) toolbox v20b [20]. The rs-fMRI data preprocessing included removal of the first 10 functional images to reduce the initial image inhomogeneity, slice timing correction, coregister, and normalization into MNI space using transformations from segmentation. Then, images were resampled to 3 × 3 × 3 mm^3^ and smoothed with a 6 mm FWHM isotropic Gaussian kernel. Subsequently, images were then bandpass filtered to 0.008–0.09 Hz, and outlier scans were identified by artifact detection tools (ART). In addition, regression of the six motion parameters and their first-order derivatives, regression of white matter (WM) and cerebrospinal fluid (CSF) signals following the implemented CompCor strategy, and detrending were further included [21].

The seed region of the bilateral EC was defined using Anatomy toolbox v2.2c [22]. The correlation coefficients between the seed voxels and the other brain voxels were calculated in all participants. To improve the normality, the correlation r value was converted to a z-value using Fisher’s r-to-z transformation [23].

### 2.5. Statistical Analysis

The statistical software SPSS version 22.0 was used to analyze clinical and neuropsychological data. A Kolmogorov-Smirnov (KS) test was adopted for all data in order to choose parametric or nonparametric tests. Kruskal–Wallis testing (for non-parametric test) or one-way analysis of variance (ANOVA; for parametric test) was used to comparing the four groups, followed by Bonferroni’s post hoc test for comparing between groups at a significance level of *p* < 0.05.

Whole brain voxel-wise regression analysis was performed to estimate the relationship between the olfactory performance and the EC-FC values in 82 PD patients. Results were thresholded based on an uncorrected voxel-wise height threshold of *p* < 0.001 combined with an FWE-corrected cluster-wise threshold of *p* < 0.05. Regions with significant correlations were defined as regions of interest (ROIs), and FC values of these ROIs were extracted for one-way analysis of covariance (ANCOVA) with SPSS to demonstrate differences in EC-FC strength among PD-normal olfaction, PD-mild macrosomia, PD-severe macrosomia, and PD-anosmia groups. Post hoc comparisons were performed using a two-sample *t*-test to determine group differences. Furthermore, Pearson correlation analysis was performed to investigate the relationship between FC values of these ROIs and other neuropsychological assessments.

In addition, regression models were used to evaluate risk factors for the decreased olfactory function. When the *p* value of the variable was less than 0.05 on the univariate analysis, it entered into the multivariate analysis. A value of *p* < 0.05 in the multivariate analysis was considered an independent risk factor of PD. In multiple linear regression, all the variance inflation factors are less than 10. There is no collinearity between the predictive variable [24].

## 3. Results

### 3.1. Demographic and Neuropsychological Results

Demographic characteristics as shown in Table 1, PD patients in the four groups were matched in age, gender, education, and disease duration (*p >* 0.05). Kruskal Wallis test revealed significant differences in GDS (*p* = 0.047) and UPSIT (*p* < 0.001). Post hoc comparisons showed significantly higher GDS scores in PD-anosmia compared with PD-normal olfaction (*p* = 0.007). UPSIT scores in the four groups were significantly different from each other (*p* < 0.001). No significant difference was found in the motor impairment (UPDRS-III *p* = 0.597, ADL *p* = 0.453, SCOPA-AUT *p* = 0.529) and in the remaining comparisons among the four PD groups.

### 3.2. Whole Brain Voxel-Wise Regression Analysis

**Figure 1:**
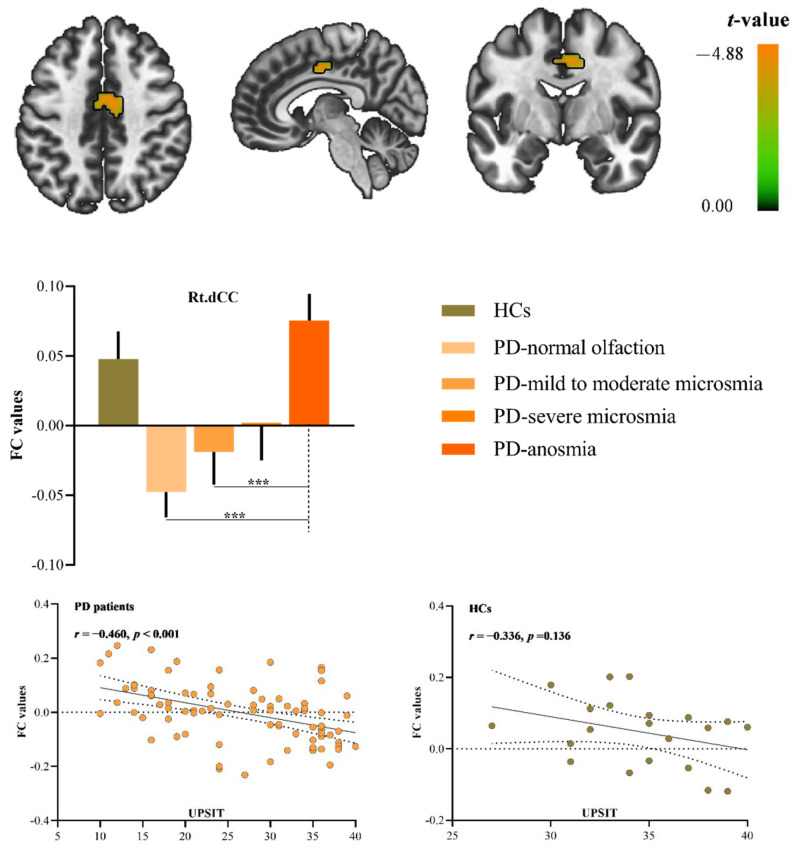
A significantly negative correlation between the olfactory performance and the FC of the EC. The scatterplots indicate a negative correlation between FC of the left EC-right dCC and UPSIT scores in all PD patients. The bar chart shows the FC of the left EC-right dCC values in the five groups. Note: The results were thresholded based on an uncorrected voxel-wise height threshold of *p* < 0.001 combined with an FWE-corrected cluster-wise threshold of *p* < 0.05. Post hoc comparisons were performed using two-sample *t*-tests to determine group differences in the extracted FC values. Abbreviations: FC, functional connectivity; EC, entorhinal cortex; dCC, dorsal cingulate gyrus; UPSIT, University of Pennsylvania Smell Identification Test; HCs, healthy controls; *** represents *p* < 0.001.

A significantly negative correlation was observed between the olfactory performance (measured by UPSIT) and the FC of the left EC in the right dorsal cingulate gyrus (dCC) in PD patients (*r* = −0.460 and *p* < 0.001, Figure 1).

One-way ANOVA analysis revealed significant differences in FC (*p* = 0.001) among PD groups with different degrees of olfactory dysfunction. Post hoc comparisons showed that PD patients with anosmia exhibited significantly higher FC values than the PD with normal olfaction or PD with mild to moderate microsomia (PD with anosmia vs. PD with normal olfaction, *p* < 0.001; PD with anosmia vs. PD with mild to moderate microsomia, *p* = 0.021, Figure 1). Meanwhile, the left EC-FC values in the right dCC were also extracted in HCs (0.048 ± 0.020). The post hoc comparisons corrected for multiple comparisons.

Except for olfactory performance, no significant correlation was detected between neuropsychological assessments and FC values.

### 3.3. Regression Models for Risk Factors Evaluation

As shown in Table 2, except for FC values of the left EC-right dCC, GDS, LNS, and STAI scores showed significant associations with the olfactory performance of PD in the correlation analysis (*p* < 0.05). Furthermore, multiple linear regression analysis found that FC values are independently associated with decreased olfactory performance (FC values: *p* < 0.001, Standardized coefficient = −0.418) (see Table 3).

## 4. Discussion

Our study aimed to detect an association between the FC of the EC and olfactory performance in early PD patients. We found a significantly negative correlation between the olfactory performance and the FC of the left EC-right dCC in PD patients. Except for olfactory performance, no significant correlation was detected between neuropsychological assessments and FC values. Further linear regression analysis revealed the GDS scores and FC of the left EC-right dCC were independent risk factors associated with olfactory performance.

The EC, as one of the primary olfactory cortices, plays a crucial role in receiving direct olfactory input from the olfactory bulb [14]. Braak reported that Lewy bodies first appear in the olfactory nerves; furthermore, Silveira-Moriyama et al. reported Lewy bodies in the primary olfactory cortex appeared very early in PD, which may cause olfactory dysfunction [25,26,27]. In the present study, a significant negative correlation was found between the olfactory performance and the FC of the left EC-right dCC in early PD patients. This finding is consistent with the results of pathological studies, suggesting that EC may be one of the earliest brain regions involved in PD. Meanwhile, we used the second-level statistics implemented in the CONN toolbox in four PD groups defining the bilateral EC as the seed regions. However, no voxel survived and corrected multiple comparisons between groups. In addition, it is noteworthy that the FC values are relatively small. These may be due to the small sample size for each PD group.

The cingulate gyrus is also engaged in multiple symptoms in PD [28]. As the projected subdivision of the secondary olfactory structure, the cingulate gyrus may affect the regulation of olfactory responses [29]. On this basis, the current finding might suggest that the cingulate gyrus can possibly be involved in olfactory dysfunction in early PD, supporting previous research as demonstrated by diffusion tensor imaging (DTI) and olfactory fMRI [30,31].

Further one-way ANOVA and post hoc analysis of the extracted FC strength revealed that PD patients with anosmia exhibited the highest EC-FC values in the dCC compared to other PD groups. Furthermore, the EC-FC values in this region in HCs (0.048 ± 0.020) also demonstrated no significant differences compared to PD patients with extracted anosmia (0.075 ± 0.020). Of note, PD patients with normal olfaction exhibited significantly lower FC values than PD patients with anosmia, and PD patients with anosmia do not differ from healthy controls. Some studies reported a complex pattern of olfactory network dysregulation in PD [7,32]. PD patients with normal olfaction exhibited the lowest FC values may suggest disrupted signal transmission as a direct effect of neurodegeneration within EC. Within olfactory information processing, there are alterations in the modulation of neuroplasticity [10]. Previous research has reported increased ReHo (Regional homogeneity) in the left ACC/PCC in PD patients with hyposmia [33]. In line with this view, in the present study, hyperactivation in PD patients with anosmia is possibly due to compensatory mechanisms. These findings might imply a compensatory mechanism that contributes to upregulated activity associated with olfactory dysfunction.

The EC and cingulate gyrus are not only important regions for olfaction but also for cognition. The potential prediction of severe hyposmia in the subsequent development of PD dementia has been reported previously [34,35,36]. However, no significant cognitive decline was identified between groups of these early PD patients, which usually occurs at the late stage of PD, which may exclude the confounding effect of cognitive status to some extent. In this context, the findings may suggest a possible biological contributor to PD’s olfactory dysfunction.

We additionally performed the correlation analysis between the other neuropsychological assessments and Lt. EC-FC values in Rt.dCC to further explore the association between these neuropsychological performance and neural activity changes in PD. As showed in the Table 2, no significant correlation was detected, which might further suggest that the current findings are less likely to be confounded by other neuropsychological assessments.

Furthermore, we conducted the multivariate linear regression analysis, and we found that the FC of the left EC-FC in the right dCC is independently associated with olfactory dysfunction. Studies show that olfactory dysfunction has a certain relationship with cognitive decline [37,38]. However, we do not find an association between cognitive decline and olfactory dysfunction. Perhaps we need more patients for further study. Longitudinal studies are needed to determine if the olfactory impairment is associated with non-motor symptoms.

There are still several limitations in this study. Firstly, although the current findings survive a rigorous threshold, the relatively small sample size may limit the generalization of this finding. Secondly, the potentially confounding effect of chronic dopaminergic medications on olfaction is required to be taken into account in further study. Thirdly, because of the heterogeneity and multiple subtypes of PD [39,40], there is not a very appropriate brain region as the control seed. In further studies, we will recruit more specific subtypes of PD patients using an appropriate control seed region. Fourthly, the FC values are relatively small. It may be due to a small sample size for each PD group. Thus, to further understand the physio-pathological mechanisms of this disease and confirm the current findings, large- scale and longitudinal cohort studies are further required.

## 5. Conclusions

The findings offer new evidence that compensatory upregulation of neural activity occurs in the brain region responsible for the olfactory information processing, which suggests that the FC characteristic may be a potential neuroimaging biomarker for early PD. Moreover, the olfactory performance combined with FC of left EC-right dCC might improve the diagnostic accuracy of early PD.

## Figures and Tables

**Table 1:** Demographic and neuropsychological characteristics.

	PD-Normal Olfaction (*n* = 26)	PD-Mild to Moderate Microsmia (*n* = 17)	PD-Severe Microsmia (*n* = 18)	PD-Anosmia (*n* = 21)	*p*
Age (years) ^a^	62.57 (10.45)	59.12 (10.18)	62.61 (10.06)	57.14 (10.18)	0.230
Gender (male/female)	18/8	11/6	12/6	14/7	0.992
Education (years)	15.19 (3.34)	15.00 (2.45)	15.78 (2.94)	15.24 (2.84)	0.754
Disease duration (months)	8.31 (9.35)	5.12 (5.59)	5.44 (5.12)	8.00 (9.48)	0.921
GDS	1.46 (1.82) *	2.41 (2.69)	2.44 (1.85)	3.62 (3.20) *	0.047
STAI	60.62 (15. 26)	66.35 (19.23)	70.72 (12.97)	68.71 (23.43)	0.269
MoCA	27.88 (1.48)	27.59 (2.09)	26.78 (1.96)	28.00 (1.41)	0.203
Modified Schwab & England ADL	91.15 (6.53)	92.65 (5.89)	89.17 (5.75)	90.95 (7.52)	0.453
MDS-UPDRS part III	20.69 (10.30)	17.59 (11.02)	22.06 (11.63)	21.52 (9.40)	0.597
JLO	12.60 (2.93)	11.81 (2.98)	11.69 (1.95)	12.39 (1.79)	0.299
HVLT-R total recall ^a^	49.54 (12.53)	48.12 (14.49)	45.61 (13.73)	44.47 (12.90)	0.584
HVLT-R recognition	49.08 (12.88)	46.18 (14.15)	43.50 (10.11)	44.76 (13.72)	0.138
LNS	12.31 (1.54)	12.18 (2.51)	10.94 (2.29)	10.71 (2.26)	0.066
Semantic fluency ^a^	55.77 (10.23)	49.12 (11.30)	54.72 (11.74)	52.48 (14.47)	0.324
SDMT	46.32 (9.64)	44.39 (10.21)	45.27 (8.75)	43.81 (11.33)	0.844
UPSIT (baseline)	36.38 (1.60) *	30.18 (1.78) *	22.06 (1.96) *	14.67 (2.60) *	<0.001
SCOPA-AUT	9.23 (5.85)	11.71 (7.58)	10.33 (5.85)	10.33 (4.18)	0.529

Note: Data are expressed as mean (standard deviation). Gender data were analyzed with χ2 test. Other *p* values were derived from the Kruskal Wallis test except for ^a^ that was derived from the independent one-way ANOVA. Bold values indicate significant differences. GDS = Geriatric Depression Scale; STAI, State-Trait Anxiety Index; MoCA = Montreal Cognitive Assessment; MDS-UPDRS = Movement Disorder Society-Unified Parkinson’s Disease Rating Scale; JLO = Benton Judgement of Line Orientation; HVLT-R = Hopkins Verbal Learning Test-Revised; LNS = Letter-Number Sequencing; SDMT = Symbol-Digit Modalities Test; UPSIT = University of Pennsylvania Smell Identification Test; SCOPA-AUT = Scales for Outcomes in Parkinson’s disease–Autonomic. * represents significant group differences in post hoc analysis.

**Table 2:** Correlations between clinical characteristics and dCC functional connectivity between the left EC and the right dCC in patients with PD.

	Olfactory Values	Statistics Values	FC Values	UPSIT Scores
Clinical Characteristics				
Age		*r*	−0.005	0.146
		*p*	0.964	0.189
GDS		*r*	0.110	−0.305
		*p*	0.325	0.005 *
STAI		*r*	0.068	−0.223
		*p*	0.544	0.044 *
MoCA		*r*	0.155	0.059
		*p*	0.164	0.594
Modified Schwab & England ADL		*r*	−0.015	0.031
		*p*	0.895	0.784
MDS-UPDRS part III		*r*	−0.045	−0.089
		*p*	0.687	0.426
JLO		*r*	−0.152	0.049
		*p*	0.173	0.66
HVLT-R total recall		*r*	0.030	0.175
		*p*	0.789	0.117
HVLT-R recognition		*r*	−0.061	0.181
		*p*	0.589	0.104
LNS		*r*	−0.170	0.288
		*p*	0.126	0.009 *
Semantic fluency		*r*	−0.124	0.044
		*p*	0.268	0.696
SDMT		*r*	0.046	0.103
		*p*	0.684	0.356
SCOPA-AUT		*r*	0.099	−0.08
		*p*	0.375	0.476

Note: Pearson correlation analysis was performed to investigate the relationship between clinical characteristics and dCC functional connectivity between the left EC and the right dCC in patients with PD. Abbreviations: GDS = Geriatric Depression Scale; STAI, State-Trait Anxiety Index; MoCA = Montreal Cognitive Assessment; MDS-UPDRS = Movement Disorder Society-Unified Parkinson’s Disease Rating Scale; JLO = Benton Judgement of Line Orientation; HVLT-R = Hopkins Verbal Learning Test-Revised; LNS = Letter-Number Sequencing; SDMT = Symbol-Digit Modalities Test; UPSIT = University of Pennsylvania Smell Identification Test; SCOPA-AUT = Scales for Outcomes in Parkinson’s disease–Autonomic; * *p* < 0.05.

**Table 3:** Linear regression between UPSIT and FC.

	Standardized Coefficient (B)	*p* Value	95% CI of B	
			Lower	Upper
Independent variables				
Age	0.128	0.193	−0.057	0.276
Male: 0, female: 1	−0.046	0.654	−4.61	2.950
GDS	−0.238	0.168	−2.031	0.360
STAI	0.032	0.850	−0.144	0.174
LNS	0.166	0.100	−0.132	1.469
FC of the Lt.EC-Rt.dCC *	−0.418	<0.001	−51.296	−18.045

Note: GDS = Geriatric Depression Scale; STAI, State Trait Anxiety Index; LNS = Letter-Number Sequencing; FC, functional connectivity; EC, entorhinal cortex; dCC, dorsal cingulate gyrus; * *p* < 0.05.

## Data Availability

Data used in this article were obtained from the PPMI database (www.ppmi-info.org/data, accessed on 1 December 2020).

## References

[1] Schapira A.H.V., Chaudhuri K.R., Jenner P. (2017). Non-motor features of Parkinson disease. Nat. Rev. Neurosci..

[2] Postuma R.B., Berg D. (2016). Advances in markers of prodromal Parkinson disease. Nat. Rev. Neurol..

[3] Montgomery E.B., Lyons K., Koller W.C. (2000). Early detection of probable idiopathic Parkinson’s disease: II. A prospective application of a diagnostic test battery. Mov. Disord..

[4] Marini K., Mahlknecht P., Tutzer F., Stockner H., Gasperi A., Djamshidian A., Willeit P., Kiechl S., Willeit J., Rungger G. (2020). Application of a Simple Parkinson’s Disease Risk Score in a Longitudinal Population-Based Cohort. Mov. Disord..

[5] Ponsen M.M., Stoffers D., Twisk J.W., Wolters E., Berendse H.W. (2009). Hyposmia and executive dysfunction as predictors of future Parkinson’s disease: A prospective study. Mov. Disord..

[6] Mahlknecht P., Iranzo A., Högl B., Frauscher B., Müller C., Santamaría J., Tolosa E., Serradell M., Mitterling T., Gschliesser V. (2015). Olfactory dysfunction predicts early transition to a Lewy body disease in idiopathic RBD. Neurology.

[7] Welge-Lüssen A., Wattendorf E., Schwerdtfeger U., Fuhr P., Bilecen D., Hummel T., Westermann B. (2009). Olfactory-induced brain activity in Parkinson’s disease relates to the expression of event-related potentials: A functional magnetic resonance imaging study. Neuroscience.

[8] Hummel T., Fliessbach K., Abele M., Okulla T., Reden J., Reichmann H., Wüllner U., Haehner A. (2010). Olfactory FMRI in patients with Parkinson’s disease. Front. Integr. Neurosci..

[9] Westermann B., Wattendorf E., Schwerdtfeger U., Husner A., Fuhr P., Gratzl O., Hummel T., Bilecen D., Welge-Lüssen A. (2008). Functional imaging of the cerebral olfactory system in patients with Parkinson’s disease. J. Neurol. Neurosurg. Psychiatry.

[10] Moessnang C., Frank G., Bogdahn U., Winkler J., Greenlee M.W., Klucken J. (2011). Altered activation patterns within the olfactory network in Parkinson’s disease. Cereb. Cortex.

[11] Georgiopoulos C., Witt S.T., Haller S., Dizdar N., Zachrisson H., Engström M., Larsson E.M. (2019). A study of neural activity and functional connectivity within the olfactory brain network in Parkinson’s disease. Neuroimage Clin..

[12] Biswal B., Yetkin F.Z., Haughton V.M., Hyde J.S. (1995). Functional connectivity in the motor cortex of resting human brain using echo-planar MRI. Magn. Reson. Med..

[13] Zang Y., Jiang T., Lu Y., He Y., Tian L. (2004). Regional homogeneity approach to fMRI data analysis. Neuroimage.

[14] Gottfried J.A., Zald D.H. (2005). On the scent of human olfactory orbitofrontal cortex: Meta-analysis and comparison to non-human primates. Brain Res. Brain Res. Rev..

[15] Li Y., Xu J., Liu Y., Zhu J., Liu N., Zeng W., Huang N., Rasch M.J., Jiang H., Gu X. (2017). A distinct entorhinal cortex to hippocampal CA1 direct circuit for olfactory associative learning. Nat. Neurosci..

[16] Marek K., Jennings D., Lasch S., Siderowf A., Tanner C., Simuni T., Coffey C., Kieburtz K., Flagg E., Chowdhury S. (2011). The Parkinson Progression Marker Initiative (PPMI). Prog. Neurobiol..

[17] Doty R.L., Shaman P., Dann M. (1984). Development of the University of Pennsylvania Smell Identification Test: A standardized microencapsulated test of olfactory function. Physiol. Behav..

[18] Jennings D., Siderowf A., Stern M., Seibyl J., Eberly S., Oakes D., Marek K., PARS Investigators (2014). Imaging prodromal Parkinson disease: The Parkinson Associated Risk Syndrome Study. Neurology.

[19] Fullard M.E., Tran B., Xie S.X., Toledo J.B., Scordia C., Linder C., Purri R., Weintraub D., Duda J.E., Chahine L.M. (2016). Olfactory impairment predicts cognitive decline in early Parkinson’s disease. Parkinsonism Relat. Disord..

[20] Whitfield-Gabrieli S., Nieto-Castanon A. (2012). Conn: A functional connectivity toolbox for correlated and anticorrelated brain networks. Brain Connect..

[21] Behzadi Y., Restom K., Liau J., Liu T.T. (2007). A component based noise correction method (CompCor) for BOLD and perfusion based fMRI. Neuroimage.

[22] Eickhoff S.B., Stephan K.E., Mohlberg H., Grefkes C., Fink G.R., Amunts K., Zilles K. (2005). A new SPM toolbox for combining probabilistic cytoarchitectonic maps and functional imaging data. Neuroimage.

[23] Lowe M.J., Mock B.J., Sorenson J.A. (1998). Functional connectivity in single and multislice echoplanar imaging using resting-state fluctuations. Neuroimage.

[24] Kim J.H. (2019). Multicollinearity and misleading statistical results. Korean J. Anesthesiol..

[25] Braak H., Del Tredici K., Rüb U., de Vos R.A., Jansen Steur E.N., Braak E. (2003). Staging of brain pathology related to sporadic Parkinson’s disease. Neurobiol. Aging.

[26] Braak H., Bohl J.R., Müller C.M., Rüb U., de Vos R.A., Del Tredici K. (2006). Stanley Fahn Lecture 2005: The staging procedure for the inclusion body pathology associated with sporadic Parkinson’s disease reconsidered. Mov. Disord..

[27] Silveira-Moriyama L., Holton J.L., Kingsbury A., Ayling H., Petrie A., Sterlacci W., Poewe W., Maier H., Lees A.J., Revesz T. (2009). Regional differences in the severity of Lewy body pathology across the olfactory cortex. Neurosci. Lett..

[28] Vogt B.A. (2019). Cingulate cortex in Parkinson’s disease. Handb. Clin. Neurol..

[29] Augustine J.R. (1996). Circuitry and functional aspects of the insular lobe in primates including humans. Brain Res. Brain Res. Rev..

[30] Poellinger A., Thomas R., Lio P., Lee A., Makris N., Rosen B.R., Kwong K.K. (2001). Activation and habituation in olfaction—An fMRI study. Neuroimage.

[31] Sobhani S., Rahmani F., Aarabi M.H., Sadr A.V. (2019). Exploring white matter microstructure and olfaction dysfunction in early parkinson disease: Diffusion MRI reveals new insight. Brain Imaging Behav..

[32] Su M., Wang S., Fang W., Zhu Y., Li R., Sheng K., Zou D., Han Y., Wang X., Cheng O. (2015). Alterations in the limbic/paralimbic cortices of Parkinson’s disease patients with hyposmia under resting-state functional MRI by regional homogeneity and functional connectivity analysis. Parkinsonism Relat. Disord..

[33] Lötsch J., Hummel T. (2006). The clinical significance of electrophysiological measures of olfactory function. Behav. Brain Res..

[34] Baba T., Kikuchi A., Hirayama K., Nishio Y., Hosokai Y., Kanno S., Hasegawa T., Sugeno N., Konno M., Suzuki K. (2012). Severe olfactory dysfunction is a prodromal symptom of dementia associated with Parkinson’s disease: A 3 year longitudinal study. Brain.

[35] Stephenson R., Houghton D., Sundarararjan S., Doty R.L., Stern M., Xie S.X., Siderowf A. (2010). Odor identification deficits are associated with increased risk of neuropsychiatric complications in patients with Parkinson’s disease. Mov. Disord..

[36] Morley J.F., Weintraub D., Mamikonyan E., Moberg P.J., Siderowf A.D., Duda J.E. (2011). Olfactory dysfunction is associated with neuropsychiatric manifestations in Parkinson’s disease. Mov. Disord..

[37] Ross G.W., Petrovitch H., Abbott R.D., Tanner C.M., Popper J., Masaki K., Launer L., White L.R. (2008). Association of olfactory dysfunction with risk for future Parkinson’s disease. Ann. Neurol..

[38] Masala C., Solla P., Liscia A., Defazio G., Saba L., Cannas A., Cavazzana A., Hummel T., Haehner A. (2018). Correlation among olfactory function, motors’ symptoms, cognitive impairment, apathy, and fatigue in patients with Parkinson’s disease. J. Neurol..

[39] Kalia L.V., Lang A.E. (2015). Parkinson’s disease. Lancet.

[40] Berg D., Borghammer P., Fereshtehnejad S.M., Heinzel S., Horsager J., Schaeffer E., Postuma R.B. (2021). Prodromal Parkinson disease subtypes—Key to understanding heterogeneity. Nat. Rev. Neurol..

